# Unveiling the therapeutic potential of *Lobaria* extract and its depsides/depsidones in combatting A*β*42 peptides aggregation and neurotoxicity in Alzheimer’s disease

**DOI:** 10.3389/fphar.2024.1426569

**Published:** 2024-08-13

**Authors:** Meixia Yang, Caishan Yan, Dusadee Ospondpant, Lisong Wang, Shengying Lin, Wai Lun Tang, Tina Tingxia Dong, Penger Tong, Qin Xu, Karl Wah Keung Tsim

**Affiliations:** ^1^ Division of Life Science and Center for Chinese Medicine, The Hong Kong University of Science and Technology, Hong Kong SAR, China; ^2^ Shenzhen Key Laboratory of Edible and Medicinal Bioresources, HKUST Shenzhen Research Institute, Shenzhen, China; ^3^ Department of Physics, The Hong Kong University of Science and Technology, Hong Kong SAR, China; ^4^ Kunming Institute of Botany, Chinese Academy of Sciences, Kunming, Yunnan, China

**Keywords:** Alzheimer’s disease, A*β* peptides, *Lobaria* extracts, depsides/depsidones, inhibition, disaggregation, neurite outgrowth

## Abstract

**Background:** The development of effective inhibitors that can inhibit amyloid *β* (A*β*) peptides aggregation and promote neurite outgrowth is crucial for the possible treatment of Alzheimer’s disease (AD). *Lobaria* (Schreb.) Hoffm., a traditional Chinese medicine used in Himalaya region for inflammatory diseases, contains depsides/depsidones (DEPs) such as gyrophoric acid, norstictic acid, and stictic acid known for their anti-cancer and anti-inflammation properties.

**Methods:** *Lobaria* extracts were analyzed using HPLC to identify DEPs and establish standards. The inhibitory effects of *Lobaria* on A*β*42 fibrillization and depolymerization were assessed using various approaches with biophysical and cellular methods. The neuroprotective activity of *Lobaria* extracts and its DEPs aganist A*β*-mediated cytotoxicity was also evaluated.

**Results:** Norstictic and stictic acid were found in the water extract, while norstictic, stictic, and gyrophoric acid were detected in the ethanol extract of *Lobaria*. Both extracts, and their DEPs effectively inhibited A*β*42 fibrillation and disaggregate mature A*β*42 fibrils. Notably, the ethanol extract showed superior inhibitory effect compared to the water extract, with gyrophoric acid being the most effective DEPs. Additionally, herbal extract-treated A*β*42 aggregation species significantly protected neuronal cells from A*β*42-induced cell damage and promoted neurite outgrowth.

**Conclusion:** This study is the first to investigate the effect of *Lobaria* on A*β*42 and neuronal cell in AD. Given that *Lobaria* is commonly used in ethnic medicine and food with good safety records, our findings propose that *Lobaria* extracts and DEPs have potential as neuroprotective and therapeutic agents for AD patients.

## 1 Introduction

Alzheimer’s disease (AD) is the most prevalent form of dementia, which is a complex, multifactorial, progressive neurodegenerative disease primarily affecting the elderly population (Eratne et al., 2018). The complicated pathophysiology of AD is commonly attributed to excessive production and subsequent aggregation of amyloid *β* (A*β*) oligomers, which is a major cause of a series of neuronal death and tissue damage (Adlard et al., 2014). The peptide A*β* containing 39–43 amino acids is produced from sequential hydrolyzation of a transmembrane protein, the A*β* precursor, by both *β*- and γ-secretases (Hardy and Selkoe, 2002). Because of different cleavage sites of secretases, A*β* has multiple variants that have already been found in the brains of AD patients. The A*β* monomers can aggregate into insoluble fibrils having a highly ordered *β*-sheet structure. The A*β* fibril is strongly neurotoxic in human brain (Jia et al., 2018; Linse, 2019; Jia et al., 2020), and which is thus believed to be one of the main driving factors for the occurrence and development of AD (Hardy and Selkoe, 2002). Thus, the inhibition of A*β* fibrillation and disaggregation of mature A*β* fibrils are possible methods to combat AD.

In addition to A*β* fibrils-induced neuronal damage, the natural aging of neurons, characterized by decreased neuronal activities, e.g., neurite outgrowth and neuronal differentiation, presents further challenges in AD treatment. An efficient AD treatment should address not only A*β* fibrillation but also the impaired, or aged, neurons to revoke their full functionality in development and regeneration. Neurotrophic factors, such as nerve growth factor (NGF), play crucial roles in the neuronal activity by promoting processes, like neurite outgrowth (Calabrese, 2008). However, NGF is typically at low level in the brains of AD patients, and due to the blood–brain barrier, direct supplementation of NGF to human brains is not feasible (Bartus, 2000; Budni et al., 2015). Therefore, an effective drug for AD should consist of small molecules that have entry to the brain and inhibit A*β* fibrillation, disassemble mature A*β* fibrils, and promote neurite outgrowth (Chen et al., 2017; Jia et al., 2018; Linse, 2019; Jia et al., 2020). Blockage of A*β* aggregation is one of the several therapeutic strategies for AD treatment, as used in the well-known phytochemical, curcumin, and newly developed drugs aducanumab and lecanemab, in reducing A*β* plaques (Huang et al., 2023).

Natural products are valued for their bioactive compounds with minimal side effects (Pagano et al., 2020). Developing drug that can inhibit A*β* aggregation and break down mature A*β* fibrils is crucial for AD treatment. *Lobaria*, a genus of lichen, is widely used as food and/or ethnomedicine medicine to treat pneumonia in the Himalayan regions, and importantly, this herb maintains a good safety record (Yang et al., 2021). It is also a rich source of calcium, dietary fiber, and protein with a protein content as high as that in Tremella (Cui and Duan, 2000). Its metabolites, depsides and depsidones (DEPs), have antibacterial, anti-inflammatory, and cytotoxic properties (Hidalgo et al., 1994; Urena-Vacas et al., 2021). Some DEPs being found in *Lobaria*, e.g., gyrophoric acid, norstictic acid, and stictic acid, have shown effectiveness in treating cancer and inflammation (Ebrahim et al., 2016; Mohammadi et al., 2022; Yang et al., 2023). For the first time, we utilized biochemical and physical techniques to explore *Lobaria*’s inhibitory effects on A*β*42 fibrillization and depolymerization of mature fibrils. The neuroprotective potential of *Lobaria* extracts and DEPs against A*β*42-induced cytotoxicity are also investigated, suggesting *Lobaria*’s potential in AD treatment.

## 2 Materials and methods

### 2.1 Raw material preparation

The dried green-algal *Lobaria* were purchased from Yunnan market (Yunnan, China). The three DEPs, stictic acid (SA), norstictic acid (NA) and gyrophoric acid (GA), all with a purity >95%, were obtained from Cayman Chemical (Ann Arbor, MI). All chemicals and ethanol extracts were dissolved in dimethyl sulfoxide (DMSO) as the stock solutions, and controlled DMSO at 0.1% final concentration was employed during the experiments (Moskot et al., 2019). Synthetic A*β*42 was obtained from GL Biochem (Shanghai, China). Thioflavin T fluorescence (ThT) and 3-(4,5-dimethylthiazol-2-yl)-2,5-diphenyltetrazolium bromide (MTT) were acquired from Sigma-Aldrich (St. Louis, MO). Ultrapure water was prepared from a Milli-Q purification system (Millipore, Molsheim, France). All culture reagents were from Thermo Fisher Scientific (Waltham, MA). All chemicals used were of analytical grade (AR) or HPLC grade.

### 2.2 Herbal extract preparation

*Lobaria* powder (10.0 g) was dissolved in 100 mL of 90% ethanol (L_EtOH_) or distilled water (L_water_) in a 250-mL round-bottomed flask. The solutions were refluxed for 1 h and then filtered through a paper filter (Advantec, Tokyo, Japan). Then, extract was evaporated to dryness using a rotary evaporator.

### 2.3 HPLC analysis of the *Lobaria* extracts

The *Lobaria* extracts (10 mg), L_EtOH_, and L_water_, were dissolved in ethanol and analyzed by HPLC-DAD with an ACE Excel 5 Super C18 column (4.6 × 250 mm, 5 μm) (Agilent, Santa Clara, CA). The mobile phase was acetonitrile A) with 0.1% phosphoric acid B) using a gradient program of approximately 20%–80% at 0–70 min. The injection volume was 10 μL. The column temperature was at 25°C, and the absorbance was measured at 264 nm.

### 2.4 Preparation of A*β* fibrils

The synthetic A*β*42 powder was dissolved in 100% hexafluoro-isopropanol at a concentration of 1 mM to disaggregate any A*β* pre-aggregates. After sonication for 20 min at 25°C, the A*β*42 monomer solution was aliquoted and dried in a fume hood overnight for complete removal of hexafluoro-isopropanol and then stored at −20°C. To obtain A*β*42 fibrils, the dried peptide film was first resuspended in 20 μL DMSO and then dissolved in 10 mM HCl aqueous solution to obtain a monomer solution of 100 μM. The peptide film was first resuspended in 20 μL DMSO and then dissolved in 10 mM HCl aqueous solution to obtain a monomer solution of 100 μM. The aggregation kinetics of the A*β*42 monomer (100 μM) incubated at 37°C was monitored using dynamic light scattering. The A*β*42 fibril formation inclined steadily until day 3 and plateaued on day 6 of incubation (Supplementary Figure S1).

### 2.5 ThT fluorescence assay

To determine the amount of A*β*42 fibrils, the fluorescence intensity of ThT dye was measured after adding it to the protein solution, following the method by Biancalana and Koide (2010). In the inhibitory effect test, A*β*42 monomer (10 μM) was allowed to self-aggregation or co-aggregation with various concentrations of *Lobaria* extracts and DEPs, then incubated at 37°C for 6 days. For the disassembling effect, A*β* fibrils (10 μM) aged for 6 days were incubated with or without extracts and DEPs at 37°C for 5 days. ThT fluorescent dye was added at a final concentration of 20 μM, and the ThT fluorescence was measured in a 96-well black plate at excitation and emission wavelengths of 435 and 488 nm, respectively.

### 2.6 AFM measurement of the aggregate morphology

To prepare the sample for atomic force microscopy (AFM) scanning, 10 μL of each protein solution (10 μM) was deposited on a freshly cleaved mica sheet and left still for 3 min. Then, the mica surface was rinsed with about 200 μL deionized water to remove redundant protein solution and let dry for about 2 h. The dried samples were then scanned using tapping mode AFM (MFP-3D, Asylum Research, Santa Barbara, CA).

### 2.7 DLS measurement of the aggregate size

Dynamic light scattering (DLS) is a standard technique for determining the particle size in a solution, including protein aggregates. While DLS accurately measures the radius of spherical particles, it provides a more complex size measurement for non-spherical particles, like the fibrillar aggregate of A*β*42, influenced by the particle’s three-dimensional geometry. The DLS experiment utilized a 30-mW helium-neon laser (wavelength λ = 633 nm), a photomultiplier tube (Hamamatsu HC120-08), and a correlator (Correlator Flex02-12D/C), with measurements taken at a 90° scattering angle and at room temperature. The sample, consisting of 1 mL of protein solution with less than 10 μL of tested extracts or DEPs, was placed in a square quartz cuvette (length/width 1 mm). Prior to measure the auto-correlation function of the scattered light, the protein solution was gently shaken by hand and allowed to settle for about 1 min to ensure uniform dispersion of aggregates in the solution. Each measurement lasted for 30 s and was repeated 4–5 times to determine the uncertainty in the measured size.

The measured autocorrelation function of the scattered light was fitted to the second-order cumulant,

$$C\;\left(\tau \right)=1+\beta \exp\left(‐2\langle \Gamma \rangle \tau +\mu_{2}\tau^{2}\right)$$

where the decay rate *Γ* = D*q* ^2^ with *q* = 4πn sin (θ/2)/λ and D = *k_B_T/6πηR*, the scattering angle θ = π/2, the wavelength of laser λ = 633 nm, the thermal energy *k_B_T* = 4.07 × 10–21 J (*T* = 22°C), the refraction index *n* = 1.33 and the viscosity *η* = 1.0 × 10^−3^ Pas. By fitting the measured correlation function to the above equation, one could obtain the aggregate size *R*.

### 2.8 Neurite outgrowth assay

Cell differentiation was assessed based on neurite length, with a differentiated cell having a neurite longer than the diameter of the cell body. PC12 cells were seeded at low cell density in a 6-well plate (1 × 10^4^ cells/well) and cultured for 24 h. Following 24 h of serum starvation, the cells were treated with *Lobaria* extracts and DEPs, with or without a low concentration of NGF at 1.5 ng/mL, for 48 h. A positive control using NGF at 50 ng/mL was included. The morphology of PC12 cells was examined under a phase-contrast microscope (Caarl Zeiss, Oberkochen, Germany, ×10 objective), and images were captured using a digital camera (SPOT basic software, Diagnostic Instruments, MI). The neurite length of approximately 100 cells from five randomly selected visual fields was quantified using the ImageJ software.

### 2.9 Cytotoxicity assay

Rat pheochromocytoma PC12 cells from rat adrenal medulla was procured from American Type Culture Collection (ATCC 1721, Manassas, VA). These cells were cultured in Dulbecco’s modified eagle medium (DMEM), supplemented with 6% fetal bovine serum (FBS), 6% horse serum, and 1% penicillin/streptomycin (10,000 U/mL and 10,000 μg/mL). The culture was maintained in a humidified incubator with 5% CO2 at 37°C and sub-cultured until passage 20. Cell viability was evaluated using the MTT assay. PC12 cells were seeded in a 96-well plate at a density of 1 × 10^4^ cells/well and incubated for 24 h. Following a 24 h serum starvation period (1% Fetal Bovine Serum (FBS) and 1% horse serum), the cells were co-cultured with varying concentrations of *Lobaria* extracts and DEPs. To investigate the preventive effect of *Lobaria* extracts and DEPs on A*β*42 fibril-induced cell death, cells were exposed to A*β*42 aggregates (10 μM) that had been cultured for 6 days with or without *Lobaria* extracts and DEPs for 24 h in culture. For evaluating the protective role of *Lobaria* extracts and DEPs against A*β*42 fibril-induced cell toxicity, PC12 cells were pre-treated with the extracts for 24 h before being exposed to A*β*42 fibrils at 10 μM for an additional 24 h. Subsequently, the cells were treated with MTT solution (0.5 mg/mL) for another 2 h. The purple formazan produced in viable cells was dissolved in DMSO, and the absorbance was measured at 570 nm.

### 2.10 Statistical analysis

Statistical analyses were performed by using data were represented as mean ± SEM. The significance of difference was determined by one-way analysis of variance (ANOVA). The *p* < 0.05 was considered statistically significant.

## 3 Results

### 3.1 Extracts of *Lobaria* inhibit fibrillogenesis

**FIGURE 1:**
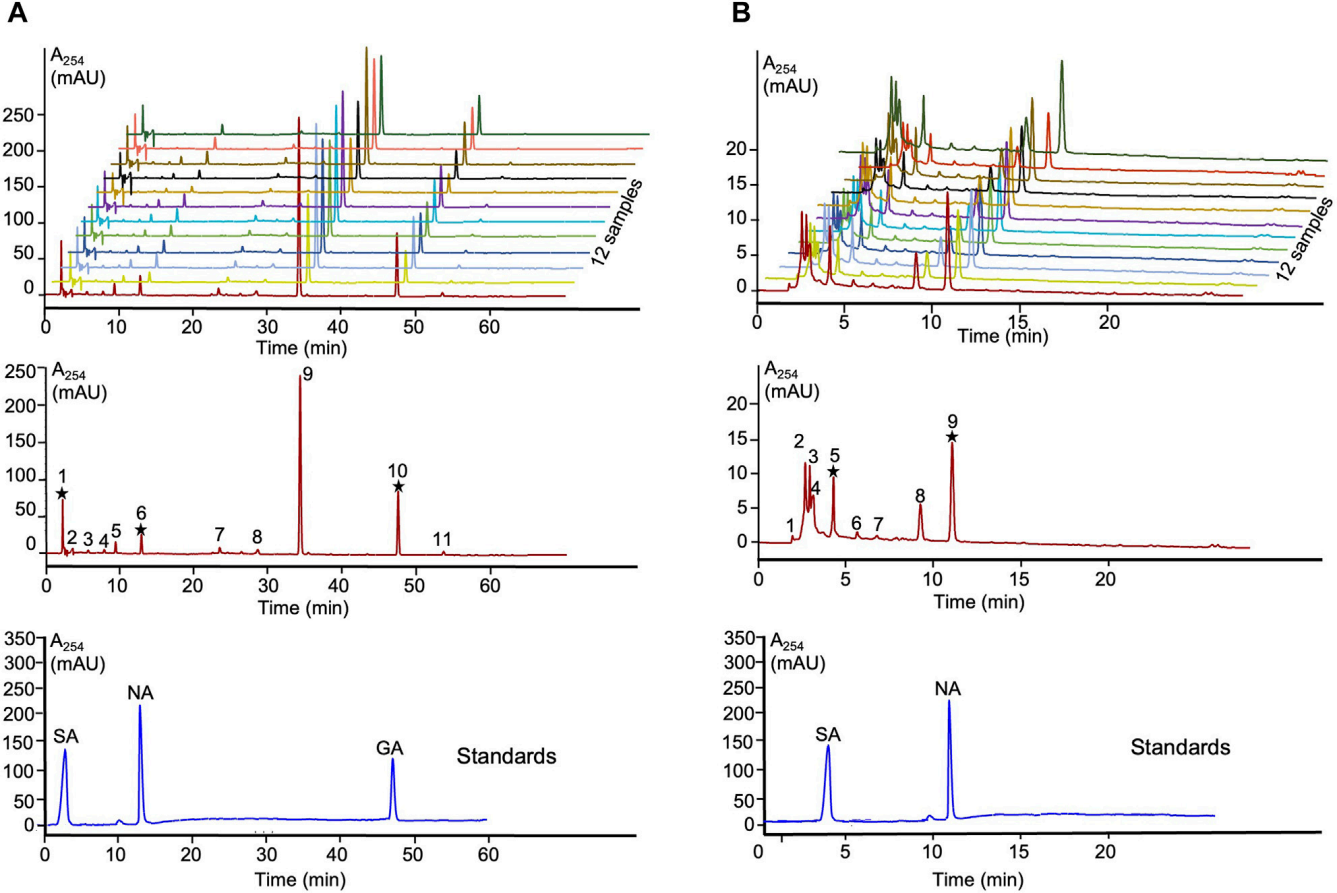
HPLC fingerprints and authentic standards of *Lobaria* extracts. **(A)** SA: stictic acid (1), NA: norstictic acid (6) and GA: gyrophoric acid (10) in L_EtOH_; **(B)** SA: stictic acid (5) and NA: norstictic acid (9) in L_water._

The obtained *Lobaria* extracts using water and 90% ethanol were labeled as L_water_ and L_EtOH_, respectively. The extraction yields were 15.3% and for L_EtOH_ and 14.5% for L_water_. Overlay chromatograms of the 12 samples at wavelength 264 nm were obtained (Figures 1A, B). A representative fingerprint of *Lobaria,* with peaks ranging from 1-11 of L_EtOH_ and 1-9 of L_water_, was utilized as common characteristic peaks for quantitative control. In conclusion, our chemical analysis confirmed the presence of only norstictic and stictic acid in L_water_, while norstictic acid, stictic acid, and gyrophoric acid were found in L_EtOH_ (Figure 1). Consistent with our previous study (Yang et al., 2023), we observed significantly higher levels of DEPs in ethanol extract compared to water extract.

**FIGURE 2:**
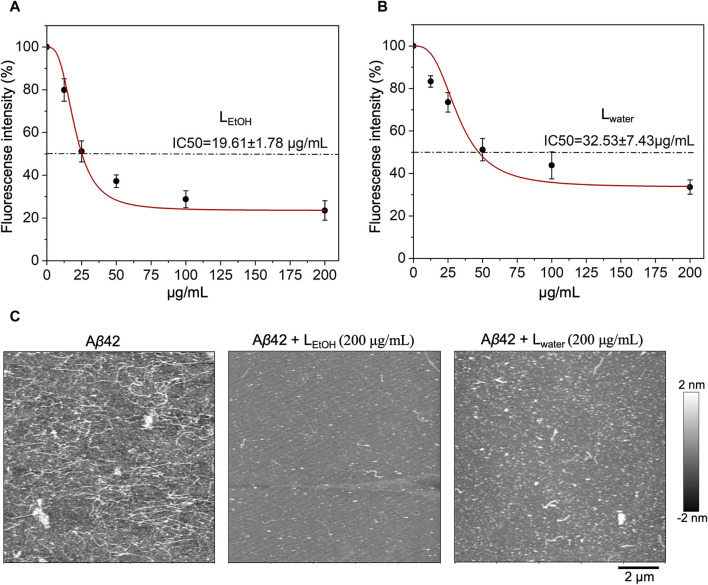
Inhibitory effect of *Lobaria* extract (L_water_ and L_EtOH_) on A*β*42 fibrillation. The half-maximal inhibitory concentration (IC50) of **(A)** *Lobaria* ethanol extract (L_EtOH_) and **(B)** water extract (L_water_) as characterized by ThT fluorescence assays. **(C)** AFM images of A*β*42 species with or without L_water_ (200 μg/mL) and L_EtOH_ (200 μg/mL). The representative data of a minimum of four independent experiments are shown in mean ± SEM fold change.

To investigate the inhibitory effect of *Lobaria* extract (L_water_ and L_EtOH_) against A*β*42 fibrillation, the ThT assay was first applied. The results, shown in Figure 2, indicated that both water and ethanol extracts of *Lobaria* inhibited A*β*42 fibrillation in dose-dependent manner. The half-maximal inhibitory concentration (IC50) for L_EtOH_ was 19.61 ± 1.78 μg/mL (Figure 2A) and for L_water_ was 32.53 ± 7.43 μg/mL (Figure 2B). When observing the A*β*42 aggregates using AFM (Figure 2C), it was found that in the presence of 200 μg/mL of either L_water_ or L_EtOH_, only short fibrils or spheroidal species were seen, instead of long fibrils that typically formed. L_water_ showed more short fibrils or spheroidal species compared to L_EtOH_, indicating that L_EtOH_ was more effective in inhibiting A*β*42 aggregation. These results show that *Lobaria* extracts can prevent the formation of long fibrillar structures during A*β*42 aggregation.

### 3.2 *Lobaria* extracts and DEPs reduce the A*β*42-induced cytotoxicity

The neurotoxicity of A*β*42 aggregates plays a crucial role in the development of AD (Kowall et al., 1991). To examine the protective activity of *Lobaria* extracts (L_water_ and L_EtOH_) on A*β*42-induced cytotoxicity of cultured PC12 cells, MTT assay was performed. Initially, the *Lobaria* extracts and three DEPs (NA: norstictic acid; SA: stictic acid; GA: gyrophoric acid) on PC12 cells was examined. At low concentration of extracts (12.5 μg/mL) and DEPs, i.e., SA (30 μM), NA (30 μM) and GA (20 μM), no adverse effects on cell survival were observed (Supplementary Figure S2). As the concentration increased, L_water_ seemed to enhance cell survival, while L_EtOH_ showed slight toxicity. The cell survival rates in different concentrations of L_water_ (25, 50, 100, 200 μg/mL) were 89.5%, 89.5%, 99.7%, and 101.5%, respectively. Meanwhile, the cell survival rates in different concentrations of L_EtOH_ (25, 50, 100, 200 μg/mL) were 97.8%, 91.5%, 91.8%, 85.7%, 80.7%.

**FIGURE 3:**
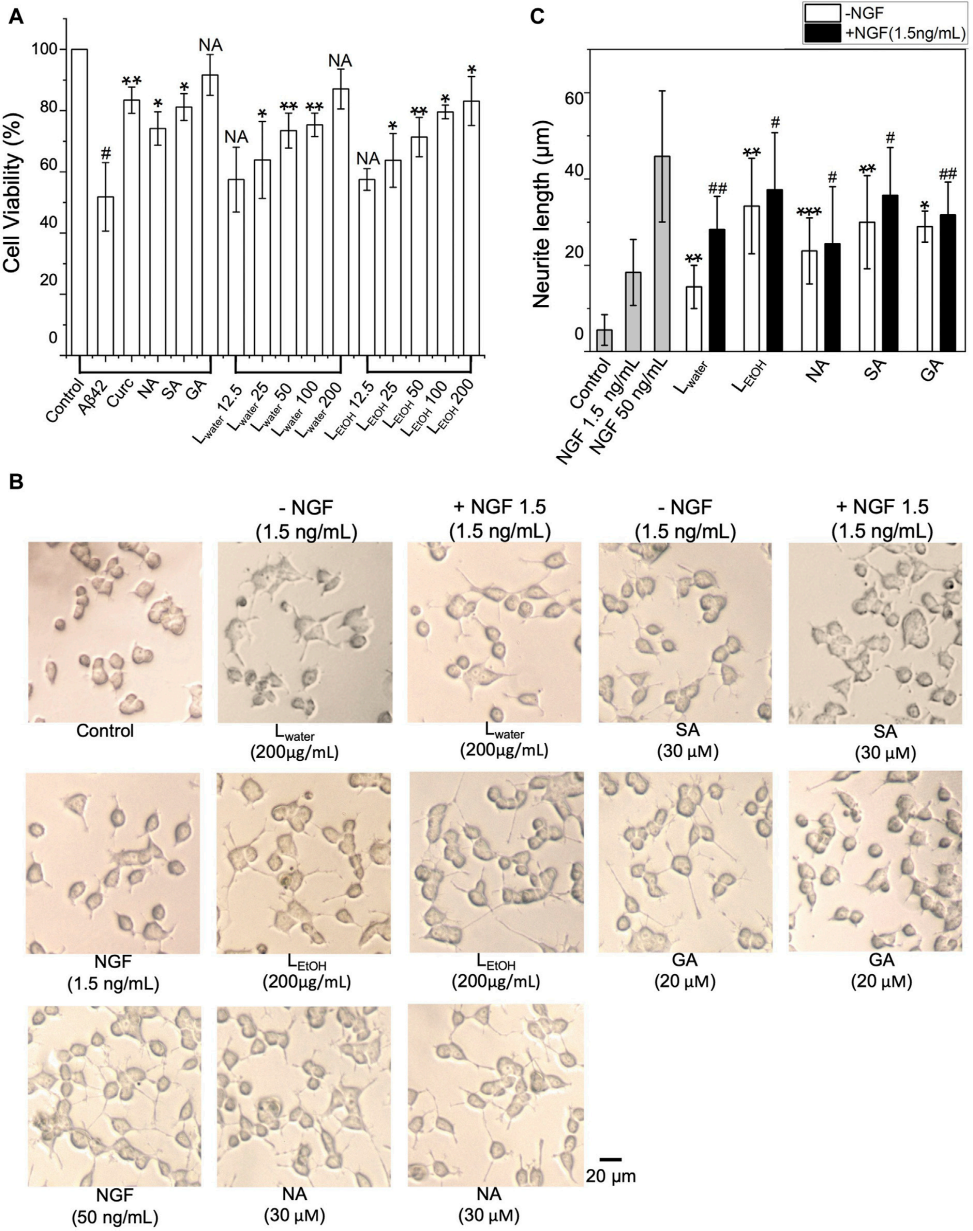
**(A)** Cytotoxicity of A*β*42 species depolymerized by DEPs (norstictic acid: NA, 30 μM; stictic acid: SA, 30 μM; gyrophoric aicd: GA, 20 µM) and different concentrations of *Lobaria* extracts (L_water_ and L_EtOH_) determined using the MTT assay. #*p* < 0.05 compared to the control group treated only with PBS buffer; NS, not significant; *p < 0.05, **p < 0.01, and ***p < 0.001 compared to the samples with A*β*42 only. Values are presented in Mean ± SEM, *n* = 4. **(B)** *Lobaria* extracts and DEPs promote neurite outgrowth. The morphology of PC12 cells was analyzed under a light microscope after 48 h treatment of 200 μg/mL L_water_, 200 μg/mL L_EtOH_ and NA (30 µM), SA (30 µM), GA (30 µM) presence or absence 1.5 ng/mL nerve growth factor (NGF). NGF at a concentration of 50 ng/mL was used as a positive control. **(C)** The average neurite length was measured after 48 h treatment with extracts and three DEPs in presence or absence of 1.5 ng/mL NGF. Values are presented in mean ± SEM fold change, *n* = 4. *p < 0.05, **p < 0.01, ***p < 0.001 compared to the control group. #*p* < 0.05, ##*p* < 0.01 compared to the group treated only with 1.5 ng/mL NGF.

We then examined whether the *Lobaria* extracts and DEPs could improve cell survival when exposed to cytotoxic A*β*42 fibrils. As shown in Figure 3A, when PC12 cells were exposed to A*β*42 fibril for 48 h, cell viability decreased by about 50%, showing the severe toxicity of A*β*42 fibrils to the cells. However, co-treating A*β*42 with *Lobaria* extracts and DEPs notably reduced the toxicity in dose-dependent manner (Figure 3A). The presence of 30 μM NA, 30 μM SA, and 20 μM GA increased cell viability by 22.3%, 29.3% and 34.8%, respectively. Moreover, the presence of 12.5, 25, 50, 100, 200 μg/mL L_water_ increased cell viability by 6.6%, 12%, 21, 6%, 23.5%, and 35.2%, respectively. Similarly, the presence of 12.5, 25, 50, 100, 200 μg/mL L_EtOH_ increased cell viability by and 5.6% 11.9%, 19.5%, 27.7%, and 31.3%, respectively, This result suggested that *Lobaria* and its DEPs effectively protected PC12 cells from the injury mediated by toxic A*β*42 species.

The impact of *Lobaria* extracts and DEPs on the neurite outgrowth in cultured PC12 cells was assessed by measuring neurite length. As shown in Figure 3B, without nerve growth factor (NGF), *Lobaria* extracts or DEPs, PC12 cells had minimal neurites with very short lengths. However, when treated with NGF, *Lobaria* extracts, DEPs or a combination of NGF with extracts or DEPs, the cells developed long neurites. The comparison of neurite length for different treatments is shown in Figure 3C. The neurite length increased significantly from about 5 μm to above 20 μm when the cells were treated with L_EtOH_ or DEPs. For the extract L_water_, the neurite length was about 15 μm. When the cells were treated with the extracts (or DEPs) and a low dose of NGF (1.5 ng/mL) simultaneously, the neurite outgrowth was further enhanced. In particular, the L_EtOH_ and SA induced neurite outgrowth almost as effectively as the high dose NGF (50 ng/mL) when in the presence of a low dose 1.5 ng/mL NGF. These results indicate that the *Lobaria* extracts and DEPs can effectively promote neurite outgrowth and thus can be very helpful in treatment of the AD.

### 3.3 *Lobaria* extracts and DEPs disassemble A*β*42 fibrils

The deposition of mature A*β*42 fibrils in the brain causes the death of neurons. The toxic oligomers are mainly formed by the secondary nucleation mechanism, catalyzed by the fibers (Cohen et al., 2013). Thus, an excellent inhibitor should not only be able to inhibit A*β* fibrillation but also be able to disaggregate mature A*β* fibrils. For example, brazilin (Du et al., 2015), tanshinone (Ren et al., 2018) and curcumin (Ono et al., 2004) have been reported to be capable of killing the fibrillar structure of A*β*42 (Yang et al., 2005; Hamaguchi et al., 2010). In this work, *Lobaria* extracts, and its DEPs were tested for their ability to disassemble A*β*42 fibrils.

**FIGURE 4:**
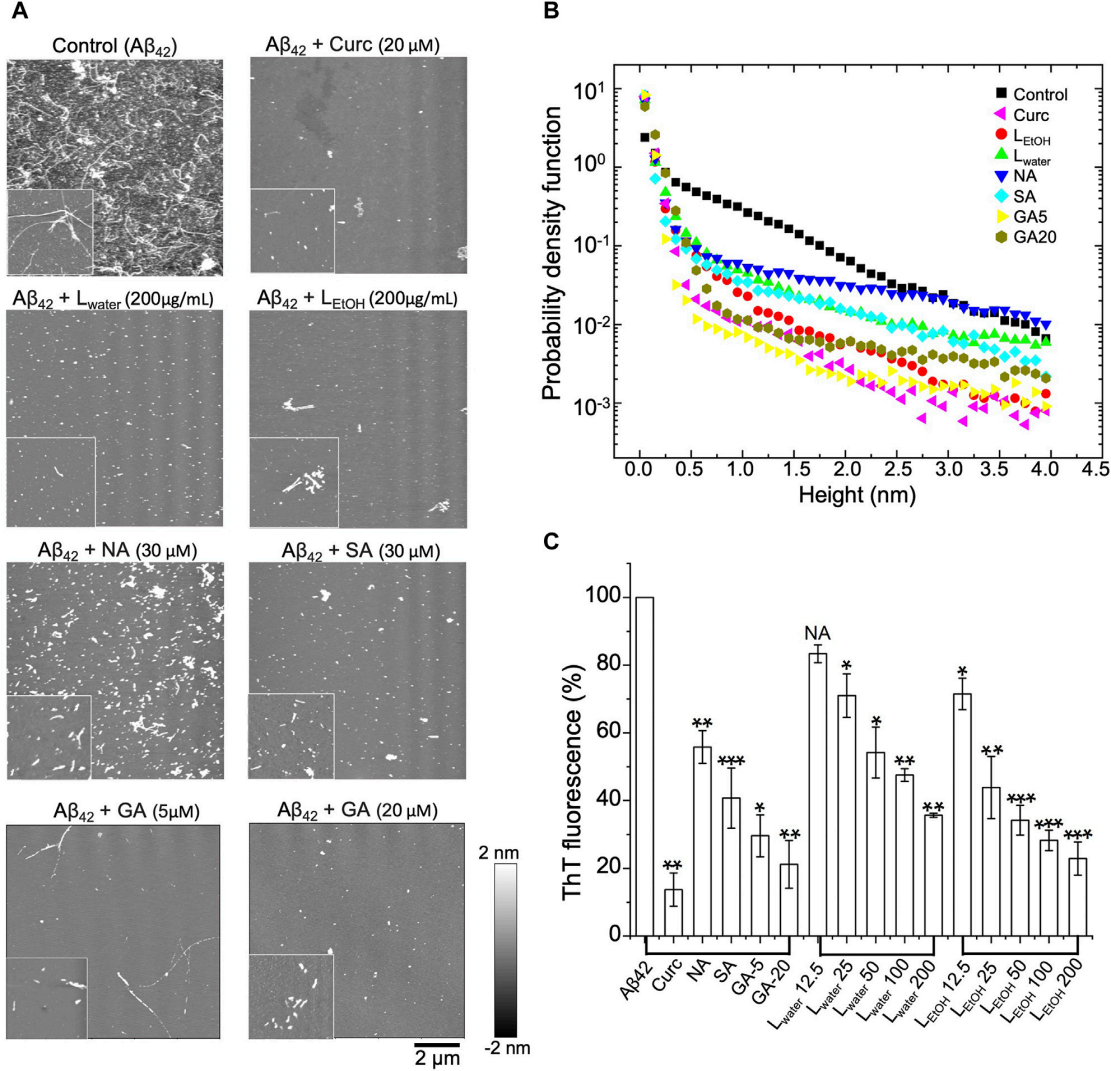
Disaggregation effect of *Lobaria* extracts (L_water_ and L_EtOH_), and DEPs compounds (norstictic acid: NA; stictic acid: SA; gyrophoric acid: GA), against A*β*42 mature fibrils, Curcumin (Curc, 20 µM) was used as a positive control. **(A)** AFM images of the aggregates after treatment of the A*β*42 fibrils with the *Lobaria* extracts or the DEPs (L_water_ 200 μg/mL, L_EtOH_ 200 μg/mL, NA 30 μM, SA 30 μM, GA 20 μM, GA 5 µM). The images have a dimension of 10 μm × 10 μm, whereas the inset images have a dimension of 2 μm × 2 µm. The representative data in four independent experiments. **(B)** Probability distributions of the height of the A*β*42 aggregates after different treatments. **(C)** The final thioflavin T (ThT) fluorescence signal of A*β*42 mature fibrils incubated with NA, SA, GA and different concentrations of L_water_ and L_EtOH_ for 3 days. Values are presented in mean ± SEM fold change. *n* = 4, NS, not significant; *p < 0.05, **p < 0.01, and ***p < 0.001 compared to the samples with A*β*42 only.

We used AFM to observe the structure of A*β*42 aggregates with and without treatment of *Lobaria* extracts (L_EtOH_ and L_water_), and its DEPs (norstictic acid: NA; stictic acid: SA; gyrophoric acid: GA). As shown in Figure 4A, the untreated A*β*42 contained a dense population of mature fibrils with each fiber being several micrometers long. Once the A*β*42 fibrils solution was treated with L_water_ (200 μg/mL), L_EtOH_ (200 μg/mL), NA (30 μM), SA (30 μM), GA (20 μM), and curcumin (Curc, 20 μM), the long fibrils disappeared, and the remains were mostly spheroidal species, which could be the oligomers or oligomer clusters. Importantly, it was found that the A*β*42 remains contained a few short fibers, as shown by the insets of Figure 4A. Thus, the A*β*42 fibrils were broken down into oligomers or short fiber fragments by the *Lobaria* extracts or DEPs. The distributions of the height and length of the treated and untreated A*β*42 aggregates are shown in Figure 4B; Supplementary Figure S3, respectively, which statistically displayed the disaggregation effect. However, due to the significant uncertainties in preparing dried protein samples and the limited imaging area, the relative effectiveness of the extracts and DEPs could not be resolved.

To have a quantitative comparison of the disaggregation effect of different treatments, the ThT assay was applied. In the experiment, the mature A*β*42 fibrils were first co-incubated with L_water_, L_EtOH_, SA, NA, GA and curcumin, respectively, for 3 days. After that, ThT fluorescence dye was added to the solution, and the fluorescence intensity was measured. The relative intensity for different treatments compared to the control (i.e., PBS) was shown in Figure 4C. It was found that all treatments reduced the ThT fluorescence intensity, which signified a reduction of the *β*-sheet structure of the aggregates. The two extracts, L_EtOH_ and L_water_, showed an improved efficiency with increasing concentrations, but L_EtOH_ reduced the aggregates more than L_water_ at all concentrations. For the three DEPs, the reduced efficiency rank was: GA > SA > NA, given about the same concentration of the DEPs. These results further confirmed the AFM results that the *Lobaria* extracts, and its DEPs, disassembled the mature A*β*42 fibrils by killing the *β*-sheet structure.

**FIGURE 5:**
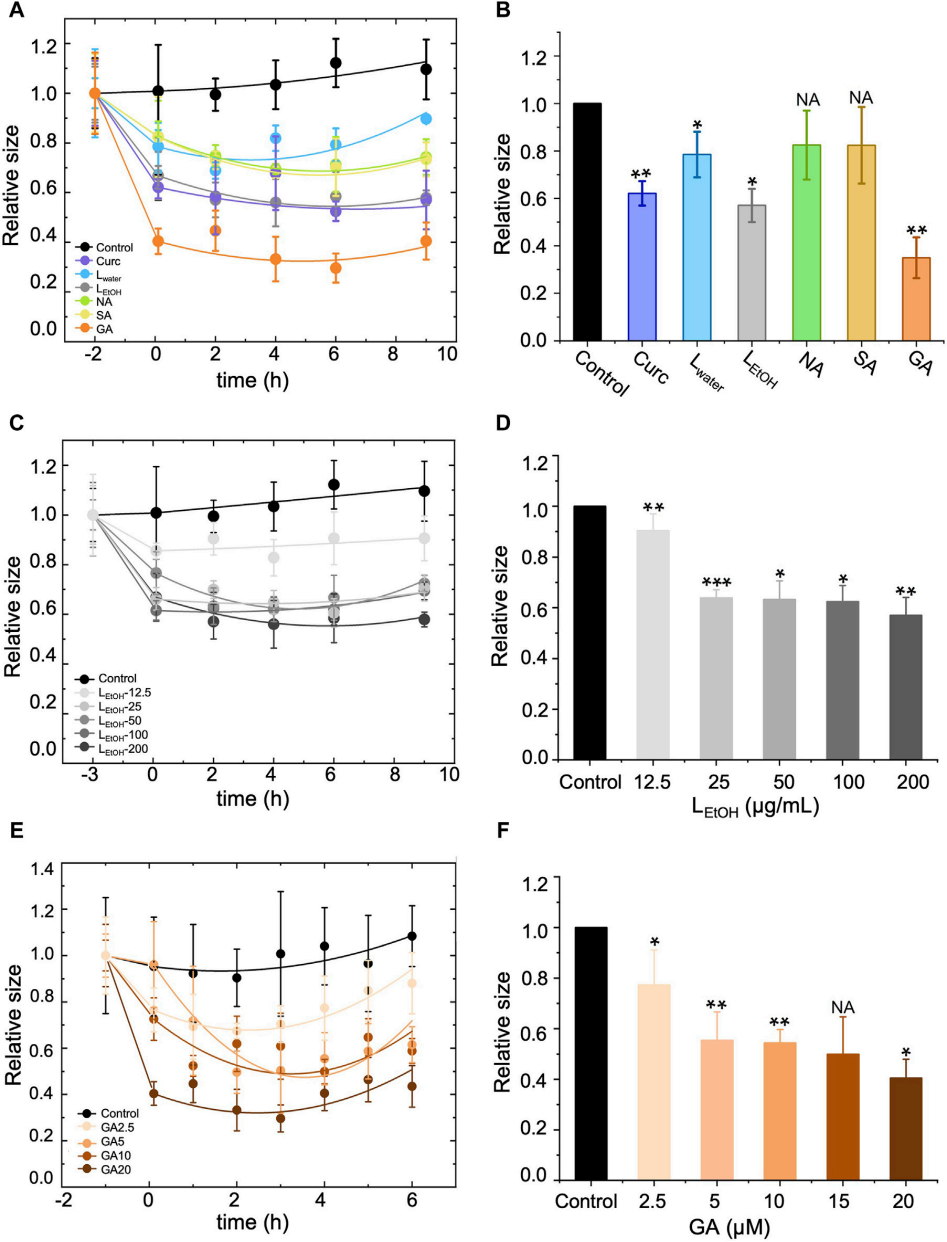
Effect of different treatments, i.e., *Lobaria* extracts (L_water_ and L_EtOH_), norstictic acid (NA), stictic acid (SA), gyrophoric acid (GA) and curcumin (Curc) on the disassembly of the A*β*42 mature fibrils by dynamic light scattering assay (DLS). Curcumin (Curc, 5 μg/mL) was used a positive control. **(A)**, **(C)** and **(E)** show change of the aggregate size with time for treatment of, respectively, **(A)** different materials (200 μg/mL L_water_, 200 μg/mL L_EtOH_, 30 μM NA, 30 μM SA, 20 μM GA, 5 μM Curc), **(C)** different concentrations of L_EtOH_ (12.5, 25, 50, 100, 200 μg/mL), and **(E)** different concentrations of GA (2.5, 5, 10, 20 μM). The first data point of each curve was measured right before the addition of the extracts or DEPs and thus, represents the original size of the A*β*42 aggregates (mature fibrils). After the treatment, the aggregate size decreased with time and eventually saturated. **(B)**, **(D)** and **(F)** show a comparison of the aggregate size at time = 2 h after the treatment of, respectively, **(B)** different materials (200 μg/mL L_water_, 200 μg/mL L_EtOH_, 30 μM NA, 30 μM SA, 20 μM GA, 5 μM Curc), **(D)** different concentrations of L_EtOH_ (12.5, 25, 50, 100, 200 μg/mL), and **(F)** different concentrations of GA (2.5, 5, 10, 20 μM). Values are presented in mean ± SEM fold change. *n* = 4, NS, not significant; *p < 0.05, **p < 0.01, and ***p < 0.001 compared to the samples with A*β*42 only of control.

We conducted DLS assays to measure the size of A*β*42 aggregates in solution over time when treated with L_water_ (200 μg/mL), L_EtOH_ (200 μg/mL), NA (30 μM), SA (30 μM), GA (20 μM), and curcumin (5 μM). The results are shown in Figure 5A. In the figure, the data point at time 0 h was obtained right after (within 5 min) addition of the extracts or DEPs, and the data for the aggregate size right before the treatment was shifted to time −2 h for clarity. The measured aggregate size was normalized by the one right before the treatment. The aggregate size showed a remarkable decrease within 2 h of treatment for all tested extracts or DEPs and remained almost unchanged thereafter (Figure 5A). This result suggests that the *Lobaria* extracts and DEPs disassembled the A*β*42 fibrils into smaller-sized structures over a short period of time (less than 2 h). However, the efficiency in reducing the aggregate size varied. We plotted the relative aggregate size measured at time 2 h for all the treatments in Figure 5B, which showed that the rank of aggregate reduction efficiency was gyrophoric acid > L_EtOH_ > curcumin > stictic acid ≈ norstictic acid ≈ L_water_. Furthermore, we examined the concentration-dependence effect on aggregate reduction for the most effective treatments, L_EtOH_ and GA (Figures 5C–F). The results showed that the aggregate reducing efficiency increased with increasing concentrations of L_EtOH_ (Figures 5C, D) and GA (Figures 5E, F) and saturated at a critical concentration, above which there was no further improvement. The critical concentrations of L_EtOH_ and GA were 25 μg/mL and 5 μM respectively, corresponding to a reduction efficiency of ∼40% and ∼50%.

## 4 Discussion

The inhibitors having properties of inhibiting A*β* aggregation and promoting neurite outgrowth are novel drugs for the treatment of AD. In our study, we discovered that *Lobaria* water and ethanol extracts (L_water_, L_EtOH_) exhibit a superior inhibitory effect on A*β* aggregation through systematic biophysical and cellular experiments. The ThT fluorescence assay demonstrated that L_water_ and L_EtOH_ effectively delayed the onset of A*β*42 fibril formation and ultimately suppressed the formation of *β*-sheet-rich A*β*42 aggregates, with IC50 values of 19.61 ± 1.78 μg/mL and 32.53 ± 7.43 μg/mL, respectively (Figure 2). Increasing the concentrations of L_water_ and L_EtOH_ to 200 μg/mL resulted in significant inhibition of A*β* fibril formation. Notably, while L_water_ exhibited a cell survival rate-promoting effect with increased concentration, L_EtOH_ showed weak toxicity. Therefore, utilizing low concentrations of L_EtOH_ for therapeutic purposes may be beneficial, as it demonstrates good functionality. AFM experiments further proved that L_water_ and L_EtOH_ could suppress the formation of long and dense A*β*42 such that only short fibrils and amorphous species of the A*β* aggregate could be formed. At the same time, MTT and neurite outgrowth tests have been performed and showed that these herbal extract-treated A*β* aggregation species have low toxicity and thus significantly protect neuronal cells from A*β*-induced cell damage (Figure 3). Within the experimental concentration range of L_water_ and L_EtOH_, higher L_water_ and L_EtOH_ doses showed less toxicity of the A*β* species (Supplementary Figure S2).

What is more interesting is that A*β*42 mature fibrils can be depolymerized into short stick or amorphous off-pathway species with low toxicity in the presence of different amounts of L_water_ and L_EtOH_, along with DEPs (Figure 3A). Among the extracts, L_EtOH_ outperforms L_water_, with a high concentration of L_EtOH_ (200 μg/mL) showing a disaggregating effect similar to that of 5 μM curcumin. Among the DEPs, gyrophoric acid demonstrates the highest efficacy, as 20 μM gyrophoric acid disassembled A*β*42 mature fibrils more effectively than 30 μM stictic acid, 30 μM norstictic acid, and 5 μM curcumin (Figures 5A, B).

The superior effectiveness of L_EtOH_ over L_water_ in inhibiting A*β* aggregation, disaggregating A*β* mature fibrils and promoting neurite outgrowth could be attributed to the higher content of DEPs in L_EtOH_, especially the gyrophoric acid. The symbiotic relationship between fungus and a photosynthetic partner in lichens produces a distinct metabolism that leads to the production of complex secondary metabolites, such as DEPs. Many DEPs have exhibited pharmacological properties, such as antioxidant, antimicrobial, and cytotoxic activities (Urena-Vacas et al., 2021). Most studies on DEPs have been conducted in cell models, with limited *in vivo* studies and clinical trials. Our study, for the first time, revealed the strong inhibitory effects of three DEPs (NA, SA, GA) on A*β*42 fibrillation and disaggregation of mature fibrils, with GA showing the highest effectiveness (Figures 5A, B). Furthermore, a molecular docking analysis was conducted to investigate the interactions between A*β* peptides and GA. The results showed a binding energy of −15.1 kJ/mol for GA, whereas curcumin had a binding energy of −22.4 kJ/mol (Supplementary Figure S4). According to molecular docking analysis, curcumin was found to bind to A*β* protein by forming a key hydrogen bond with ALA21 residue (Supplementary Figure S4B). On the other hand, GA displayed significant binding affinity to A*β* and bound to the same domain as curcumin. Interestingly, it was observed that GA established three hydrogen bonds with LEU17, PHE19 and ALA21, respectively (Supplementary Figure S4A). This suggests that GA may exhibit strong binding activity at the binding site, potentially leading to higher inhibitory effects on A*β* fibril formation. These findings align with our experimental results demonstrating the positive impact of GA. As a biological polysaccharide, GA has good bioavailability, which has also been proven by previous studies (Sovrlic et al., 2023; Thakur et al., 2023).

While *Lobaria* extracts and DEPs show promise as effective inhibitors of A*β* aggregation and potential neuroprotective agents for AD treatment, their development into anti-AD drugs poses significant challenges. *Lobaria* and other DEP-producing lichen species have slow growth rates, limiting DEPs productivity. To overcome limitations, alternative methods, like artificial culturing or fermentation, are being explored to acquire essential active ingredients and decrease dependence on natural resources. In addition, it is important to note that while the cell study provides valuable insights, further validation through a series of *in vivo* studies is essential for generalization. To overcome the challenges associated with *in vivo* efficacy and safety testing, the key considerations, such as selecting appropriate animal models, determining optimal dosage for *in vivo* administration, conducting comprehensive toxicity studies, assessing behavioural effects, should be implemented.

## 5 Conclusion

In conclusion, *Lobaria* extracts, and its DEPs demonstrate significant potential as inhibitors of A*β*42 peptides aggregation and neurotoxicity in AD. The study highlights the superior inhibitory effects of *Lobaria* water and ethanol extracts (L_water_, L_EtOH_) on A*β*42 fibrillation through comprehensive biophysical and cellular experiments. Notably, the extracts effectively delayed the onset of A*β*42 fibril formation and inhibited the formation of *β*-sheet-rich A*β*42 aggregates. Furthermore, the DEPs, particularly gyrophoric acid, exhibited strong efficacy in disintegrating mature A*β* fibrils, offering promising neuroprotective effects. The discoveries made here open up possibilities for the future utilization of *Lobaria* extracts and DEPs in the treatment of AD, offering a diverse range of therapeutic advantages.

## Data Availability

The original contributions presented in the study are included in the article/Supplementary Material, further inquiries can be directed to the corresponding author.

## Abbreviations

Aβ, amyloid β peptides; AD, Alzheimer’s disease; DEPs, depsides and depsidones; NGF, nerve growth factor; ThT, thioflavin T fluorescence; AFM, atomic force microscopy; DLS, dynamic light scattering; MTT, 3-(4,5-dimethylthiazol-2-yl)-2,5-diphenyltetrazolium bromide; LEtOH, Lobaria ethanol extract; Lwater, Lobaria water extract; FBS, fetal bovine serum; DMSO, dimethyl sulfoxide; GA, gyrophoric acid; NA, norstictic acid; SA, stictic acid.
