# Supplementary material for: Unveiling the therapeutic potential of Lobaria extract and its depsides/depsidones in combatting Aβ42 peptides aggregation and neurotoxicity in Alzheimer’s disease
Source: Front Pharmacol. 2024 Aug 13;15:1426569. doi: 10.3389/fphar.2024.1426569 (PMC11347406; doi:10.3389/fphar.2024.1426569)
Supplement: Supplementary file 1 [file DataSheet1.docx]

Supplementary Material

# Supplementary Figures


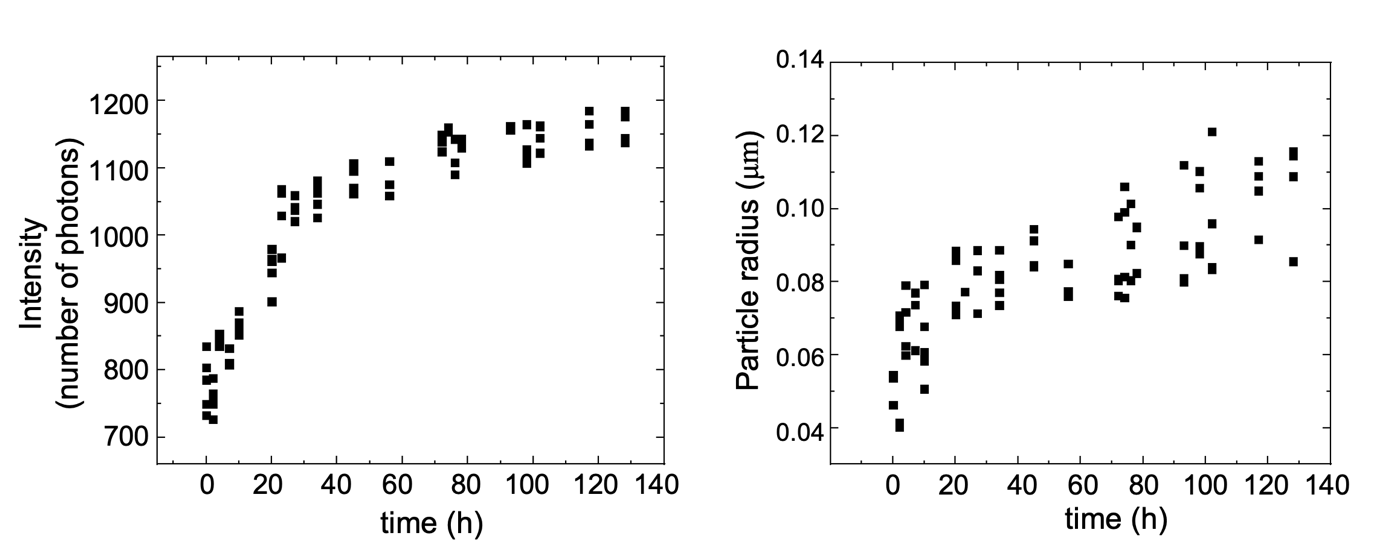


**Supplementary Figure 1.** The aggregation of the A*β*42 monomer (100 μM) incubated at 37°C for 6 days was analyzed using the dynamic light scattering (DLS) assay. Left: Intensity of the scattered light as a function of time. Right: Average aggregate size as a function of time.


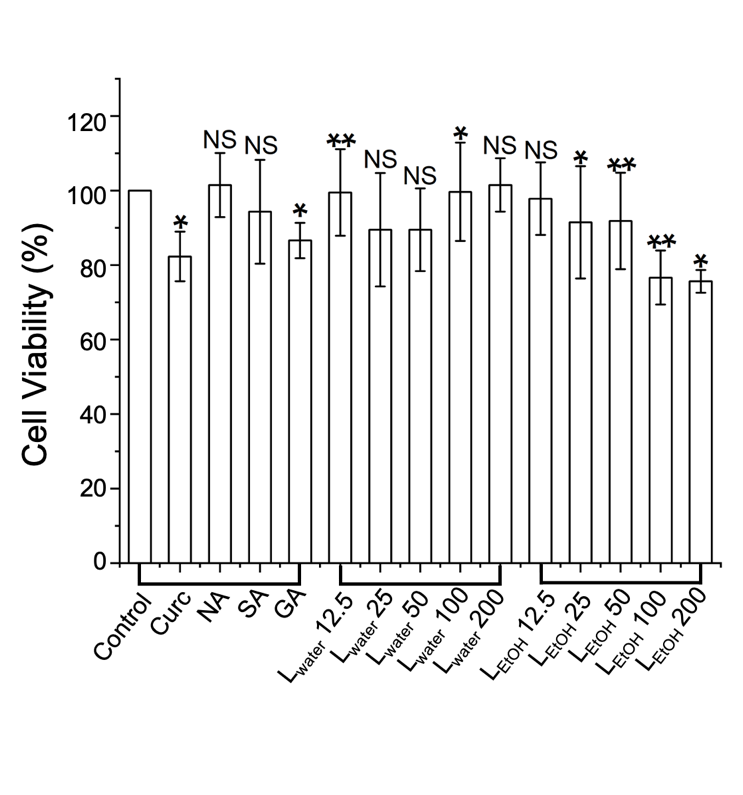


**Supplementary Figure 2.** (A) The effect of *Lobaria* extracts and its DEPs on the growth of PC12 cells. PC12 cells were cultures for 48 h in the absence or presence of different doses of extracts (0-200 µg/mL) and norstictic acid (NA; 30 µM), and stictic acid (SA; 30 µM), gyrophoric acid (GA; 20 µM) as indicated, and cell growth was measured using clorimetric MTT. NS, not significant; *p < 0.05, **p < 0.01, and ***p < 0.001 compared to the samples of control.


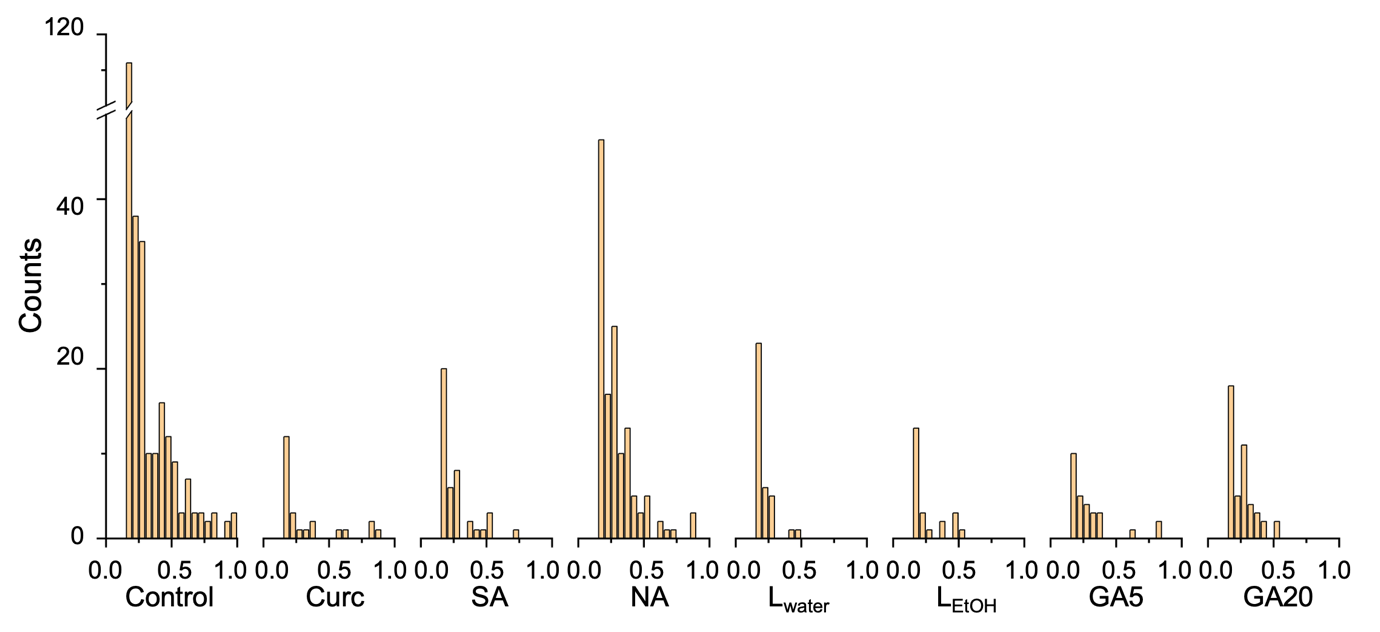


**Supplementary Figure 3.** A*β* aggregate length of different drugs (curcumin 20 µM, L_water_ 200 μg/mL, L_EtOH_ 200 μg/mL, norstictic acid (NA; 30 µM), and stictic acid (SA; 30 µM), gyrophoric acid (GA; 5 and 20 µM) based on AFM images.


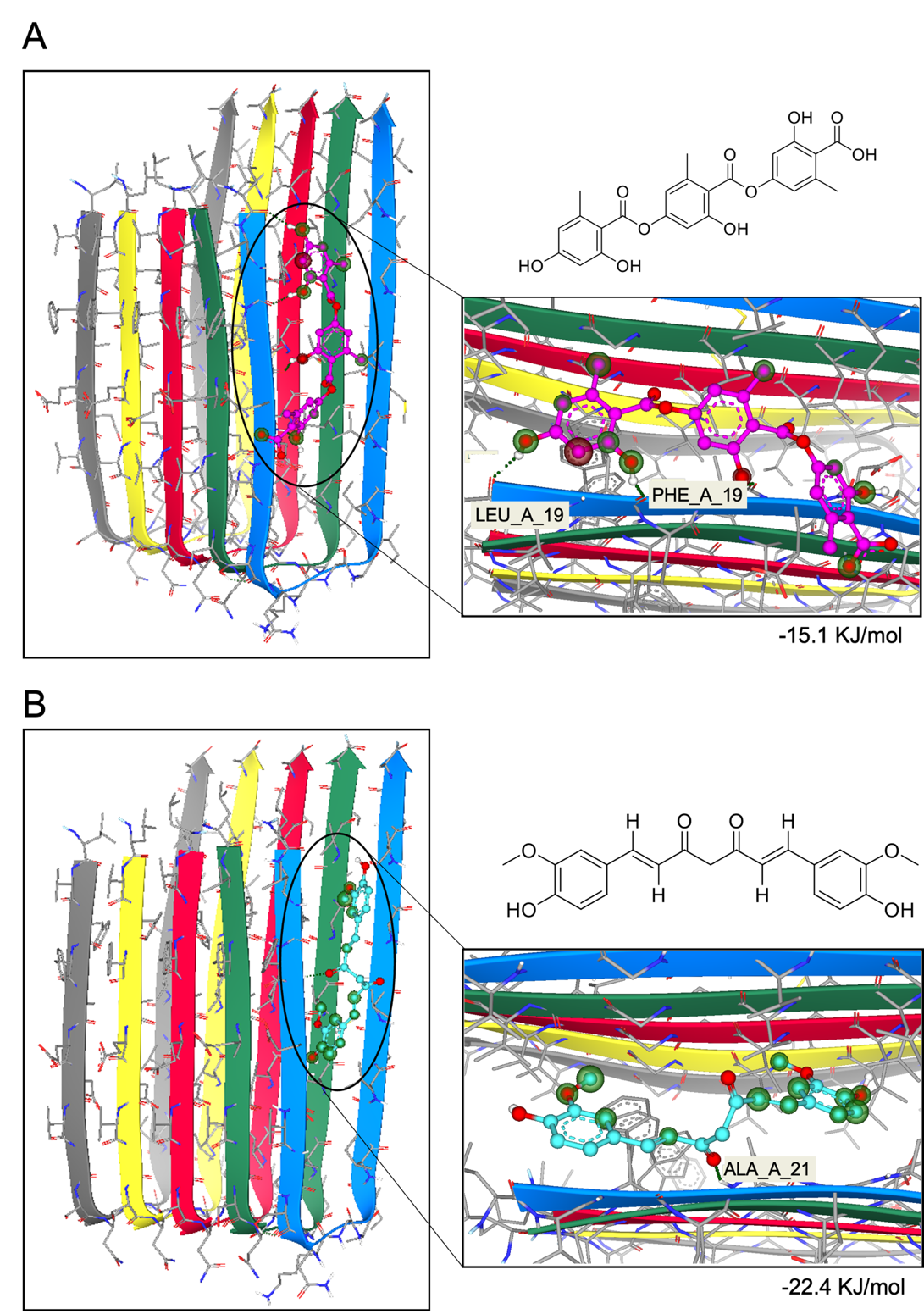


**Supplementary Figure 4.** Docking image of gyrophoric acid (GA) (A) and curcumin (B) against Amyloid beta peptide (2BEG). Positive control: Thioflavin T (-18.9 KJ/mol), AZD2184 (-20.5 KJ/mol).
